# Prevalence of human papillomavirus (HPV) in oral cavity and oropharynx

**DOI:** 10.1016/S1808-8694(15)30068-9

**Published:** 2015-10-19

**Authors:** Therezita Peixoto Patury Galvão Castro, Ivo Bussoloti Filho

**Affiliations:** a Postgraduate student (PhD) - Department of Otorhinolaryngology - FCMSCSP, Assistant Professor of Otorhinolaryngology - UNCISAL and UFAL.; b PhD in Medicine (Otorhinolaryngology), Professor of Otorhinolaryngology- FCMSCSP.

**Keywords:** human papillomavirus (HPV), oral

## Abstract

The prevalence of human papillomavirus (HPV) in the oral cavity and oropharynx has not yet been as well studied as its infection of the vaginal tract. However, new study are emerge after the development of molecular biology techniques. The objective of this study is to show the prevalence of HPV in the oral cavity and the oropharynx. An ample bibliographic review was done showing a prevalence of HPV 6, 11 in a normal oral mucous membrane (latent infection). In oral benign lesions associated with HPV, a prevalence of HPV 6 and 11 was observed in squamous cell papilloma (SCP) and condylomas acuminatum, while HPV 2 and 57 were more prevalent in verruca vulgaris lesions. As for focal epithelial hyperplasia (FEH) and oral cancer, especially squamous cell carcinoma (SCC), the prevalence was of HPV 13 and 32, and HPV 16, respectively. The last findings are, nonetheless, controversial. The last findings are, nonetheless, controversial. Showed also discrepancy result the prevalence of human papillomavírus (HPV) in normal oral mucous (latent infection) and in oral cancer, however evidenced confirmatory result in oral benign lesions associated with virus.

## INTRODUCTION

HPV is the acronym used to identify the human papillomavirus, responsible for the condiloma acuminata (from the Greek term kondilus = round tumor and the Latin term acuminare = make it pointed)^1^.

Human papillomavirus belong to a large family of viruses, the papovaviridae. They are small (about 55nm in diameter) and epitheliotropic. Their genome is made up of 7,200 - 8,000 base pairs with molecular weights of 5.2 x 106 Daltons. They have a capsule with 72 capsomeres of icosahedral structures, without lipoprotein envelope, and in a single circular double DNA molecule^2^, ^3^, ^4^, ^5^.

Human papillomavirus infections and its spread occur all over the world. HPV infect the skin and mucosas and might induce the formation of both benign and malignant tumors6. The infection starts when the virus penetrates the new host, through micro-injuries. The development of this incubation phase into active expression depends on three factors: cell permeability, virus type and the host’s immune status^7^.

HPV prevalence in the normal oral mucosa (latent infection) and its relation to oral cancer have generated conflicting opinions. The discrepancy observed is mainly attributed to a variation in the sensitivity of the methods employed and epidemiologic factors related to the group of patients examined^8^.

The goal of the present study is to carry out a bibliographic review on the prevalence of human papillomavirus in the oral cavity and oropharynx through HPV detection methods, immunohistochemistry, in situ hybridization, Southern blot hybridization and polymerase chain reaction, checking the HPV types prevalent in the normal oral mucosa (latent infection), in benign oral and oropharynx lesions (squamous cells papilloma, condiloma acuminata, common wart and focal epithelial hyperplasia), and in oral cancer.

## LITERATURE REVIEW

### HPV detection methods

The diagnosis of human papillomavirus in the oral mucosa may be suspected when one inspects the lesion, and also through cytology and biopsy. The cytological aspect of the HPV infection is characterized by:

A. Major criteria: classic coilocytes, perinuclear cytoplasmic halos and nuclear dysplasia.
B. Minor criteria: dysceratocytes, atypical immature metaplasia, macrocyte and binucleation. This method bears limited sensitivity and does not type the HPV^9^, ^10^.

The methods used to detect the HPV DNA in lesions vary broadly as far as their sensitivity and specificity are concerned^11^. These are further broken down in three categories:

Low Sensitivity: immunohistochemistry and in situ hybridization - because they only detect the virus when it is present in more than 10 copies of the viral DNA per cell.

Moderate Sensitivity: Southern blot, dot blot and reverse dot hybridizations, because they only detect the virus when present in 1 to 10 copies of the viral DNA per cell; and the High Sensitivity: PCR, because it detects the virus in less than 1 copy of the viral DNA per cell^12^.

Immunohistochemistry may detect the protein coat of HPV viral particles which are present in the lesions seen under light microscopy in paraffin bearing material or in cytological preparations, and in these cases, polyclonal antibodies are used against different types of HPV specific antigens^9^. This technique is hampered by the limited availability of antibodies against specific types of HPV, since the virus does not replicate in vitro. Commercially available antibodies are created against bovina papillomavirus capsule antigens, with an HPV crossed reaction^13^.

Hybridization tests are currently the methods of choice to detect HPV DNA or RNA in smears or tissue samples. They are made directly or after DNA and RNA amplification through PCR. The basic principle behind these hybridization techniques is the double formation between the DNA single strand, RNA molecules or cloned HPV types, denaturated DNA derivatives and viral nucleic acid molecules present in the cell, which represents the hybridization test target^10^.

The Southern blot hybridization is used in order to detect the HPV DNA from biopsies and is considered the “gold standard” to detect HPV genome. It is a sensitive and highly specific test, thus proving to be a valuable research tool, however it has no application in clinical routine tests because it takes longer and it requires harder work^10^.

Another recently developed hybridization method is the hybrid capture (HCA), which does not distinguish among the specific HPV types; and their applicability as a research method is limited, however it may represent a good test for routine clinical use^10^.

Polymerase Chain Reaction (PCR) is a technique that has revolutionized virology, because of its extremely high sensitivity^5^. It is characterized by the amplification of minute amounts of target DNA sequences in many million times. It is a cyclic thermal process that includes three stages: denaturation, in which the double DNA strand is separated in single strains; coupling, in which the primers couple specifically with their complementary single strand target-DNA sequences; and finally, the primer extension, in which a thermostable DNA polymerase generates DNA “offspring” strands which cross the region between two primers. From then on, recently generated double strands serve as models for a subsequent PCR cycle. The primers may be: type-specific primers, which detect a simple type of HPV, or the consensus primers (also called general or generic), which detect a whole array of different HPV types in a single reaction^14^.

PCR detection of HPV is usually carried out through one of the consensus primers, the MY09-MY11 or the GP5/GP6^10^, ^15^. Currently, PCR allows for a more in depth assessment of epidemiologic data, including the prevalence of latent of subclinical infections. However, it bears the following disadvantages: amplification of minute amounts of contaminating HPV DNA, which may lead to false-positive results. Each method is limited by its sensitivity, specificity, its practice, cost and its commercial availability. It is important and fundamental to assess the efficacy of different HPV detection techniques, in order to establish HPV etiology in oral lesions^10^, ^16^.

### Human papillomavirus prevalence in the oral cavity and oropharynx

HPV prevalence in the oral cavity and oropharynx is uncertain. Studies have shown dubious results, assessing a small number of patients and identifying some of the many HPV types found in mucosal lesions^17^.

So far, over 100 types of HPV have been identified^18^, ^19^. Of these, 25 types have been associated to oral lesions (HPV-1, 2, 3, 4, 6, 7, 10, 11, 13, 16, 18, 31, 32, 33, 35, 40, 45, 52, 55, 57, 58, 59, 69, 72 and 73)^20^.

In order to explain the HPV infection relationship with many oral lesions, it is important to investigate HPV prevalence in the normal oral mucosa.

### Human papillomavirus prevalence in normal oral mucosa

Papillomavirus in normal oral mucosa must be investigated throughout studies on oral cavity HPV infection natural history. Its prevalence in the normal oral mucosa is controversial^21^.

In the normal oral mucosa we have seen a great variation in HPV rates detected, from 22% to 60%^17^; from 0% all the way to 81.1% in studies using different methods, and with a limited number of individuals, and this seems to depend on the population studied and the choice of method^16^, ^21^. It has been suggested that HPV prevalence in the normal mucosa includes subclinical and/or latent infections, and that the infection with a low number of virus copies is common in the oral cavity^8^.

Table 1 depicts the techniques and the results obtained by different authors as to the identification of the HPV type in the normal oral mucosa.

**Table 1:** Results from various authors as to HPV identification in the normal oral mucosa.

Year	Investigator	Method	#P/#. C	%	HPV type
1992	Kellokoski et al.29	PCR	18/78	(23)	6, 11 e 16(83%), 18(17%)
		SB	33/212	(15)	2,6,11,13,16,18
1992	Jalal et al.27	PCR	21 /48	(44)	16
1993	Holladay and Gerald. 25	PCR	1/6	(17)	16
1995	Eike et al.16	PCR	0/61	0	-
1995	Mao et al. 34	PCR	4/26	(15)	16
1996	Tominaga et al. 76	PCR	3/3	(100)	6
		SB	0/3	0	-
1996	Cruz et al. 11	PCR	0/12	0	-
1996	Mao et al. 35	PCR	0/6	0	-
1998	D’Costa et al. 12	PCR	15/48	(31)	16
1998	Badaracco et al. 4	PCR	4/22	(18)	6(20%), 16(50%), 31, 11(10%)
1999a	Terai et al. 72	PCR	26/30	(87)	18(87%), 61(60%), 59(23%), 16(7%)
1999	Pillai et al. 51	ISH	0/10	0	-
1999	Bustos et al.8	ISH	0/33	0	-
2003	Surgyama et al. 64	PCR	16/44	(36)	16

#= number; P = positives; C = cases; ISH =in situ hybridization; SB = Southern blot hybridization; PCR = polymerase chain reaction.

Human papillomavirus prevalence in benign lesions (squamous cells papilloma, condiloma acuminata, common wart and focal epithelial hyperplasia of the oral cavity and oropharynx related to the virus).

Oral and oropharynx squamous cells papilloma are benign tumors that occur mainly between 30 and 50 years of age, although they may also occur below 10 years of age^22^. They represent about 8% of oral tumors in children^23^.

They usually involve the soft palate, tongue, frenulun linguae and the lower lip. In most of the cases, the papillomas are single and small (<1cm)^24^. They have an exophytic growth and show up both as a broad based ovoid bulging, or a pedicled lesion. The surface may present small finger-like projections, giving it a rough verruca contour. The color varies from white to pinkish, depending on the levels of keratinization and vascularization^25^.

### Oropharynx and oral condiloma acuminata

Oral condiloma acuminata is usually considered a sexually transmitted disease, acquired by oral sexual contact. Currently, the trend is to consider that the oral condiloma may be acquired not only by oral Sex, but also by self inoculation or as a result of maternal transmission^20^, ^25^, ^26^.

Clinical and histological similarities between squamous cells papilloma, condiloma acuminata and verruca in the oral cavity are clear^27^. This differentiation with PCE is difficult and purely academic. In the mouth they are usually present as small pinkish or whitish nodules, which spread in papillary projections and may be pedicled or sessile. The surface contour, in most of the cases, is cauliflower-like rather than papillomatous^24^. They occur alone or in multiple form, frequently on the tongue, lip, palate and floor of the mouth^28^.

In the genital tract, the terms papilloma and condiloma were used separately, until the year 1970; since then, both lesions are called condiloma. This modification may also be applied to oral lesions, because both the papilloma and the condiloma have the same HPV types found in genital condilomas^20^.

The human papillomavirus was detected in condilomatous oral lesions, initially by immunohistochemistry, and later by hybridization techniques, with a positiveness varying between 75% to 85% for HPV 6 and 11^20^, ^24^.

In 1991, Zeuss et al.^29^, considered HPV 6 and 11 as being the types most frequently associated to benign papillomatous oral lesions.

### Oral and oropharynx common wart

The common wart is one of the most common skin lesions, specially in children^24^. They are clinically undistinguished from PCE and condiloma, appearing as a sessile papillomatous whitish lesion of rough surface^25^. They are frequently located on the lips, hard palate, gums and tongue dorsum^30^. The diagnosis of an oral wart must be restricted to a lesion with clinical and histological characteristics of a common skin wart, and confirmed by seeing the HPV types in the skin wart^20^.

Many authors showed the presence of the virus in oral warts by immunohistochemistry and hybridization tests. They observed a variation in the HPV detection rates from 43% to 100% in oral warts^29^, ^31^, ^32^.

HPV prevalence in oral warts seems to be HPV 2, followed by the HPV 57, although more studies are necessary to identify the broad spectrum oral infectiosn caused by skin HPV types^20^, ^33^.

Table 2 shows the techniques and the results of the studies about papillomas, condilomas and warts in the oral cavity and oropharynx.

**Table 2:** Results from various studies as to HPV identification in papillomas (including condiloma and wart) in the oral cavity and oropharynx.

Year	Investigator	Lesion	Method	#. P/#. C	%	HPV type
1986	Adler-Storthz et al.^78^	Verruga	IHQ	6/11	(55)	HPV +
			ISH	6/11	(55)	2 (83%), 4 (17%)
1986	Syrjänen.^68^	Papiloma	ISH	6/7	(85.5)	6(33.5%) 11(66.5%)
			IHQ	4/7	(57)	HPV +
1986	Syrjänen. ^68^	Condiloma	ISH	2/2	(100)	6(50%) e 11(50%)
			IHQ	2/2	(100)	HPV +
1987	Eversole et al. ^18^	Condiloma	IHQ	5/20	(25)	HPV +
			ISH	18/20	(90)	6, 11 (85%), 2(5%)
1991	Zeuss et al.^81^	Papiloma	ISH	4/30	(13)	6e 11
1991	Zeuss et al.^81^	Condiloma	ISH	15/15	(100)	6e 11
			ISH	11 / 15	(73)	6, 11, 16, 18, 31, 33,35
1991	Zeuss et al.^81^	Verruga	ISH	5/5	(100)	6e 11
1991	Young et al.^79^	Papiloma	ISH	13/21	(62)	6, 11
				10 / 13	(77)	16, 18,31,33, 35
1994	Padayachee et al.^46^	Verruga	IHQ	12/21	(57)	HPV+
			ISH	15/21	(71)	2 e 57 (87%), 2 (13%)
1995	Padayachee et al.^47^	Verruga	ISH	14/19	(74)	2
			ISH	12/ 19	(63.5)	57
			PCR	8/19	(43)	2,57
1996	Tominaga et al.^76^	Condiloma	PCR	1/8	(13)	6
1998	Bishop et al.^6^	Condiloma	ISH	1/1	(100)	6e 11
1998	Badaraccoe	Condiloma	PCR	7/7	(100)	6,31,57,56,44, 16
1999b	Terai et al. ^73^	Verruga	IHQ	1/1	(100)	HPV+
			ISH	1/1	(100)	2
			SB	1/1	(100)	2

#= number; P = positives; C = cases; IHQ = immunohistochemistry; ISH =in situ hybridization; SB = Southern blot hybridization; PCR = polymerase chain reaction; HPV+ = positive human papillomavirus.

### Oral focal epithelial hyperplasia (FEH)

The term focal epithelial hyperplasia (FEH), or Heck’s disease, introduced by Archard et al.^34^ in 1965, to describe multiple nodular elevations in the oral mucosa observed in Alaska Eskimos and North and South American Indians. It is rarely observed in caucasians^35^. It has also been described in Israel, South Africa and Sweeden^36^.

It is a benign lesion that may be located in the oral mucosa, lips and tongue, and most notably in the lower lip^24^, ^37^, ^38^. It is clinically characterized by multiple painless and soft papules, of color that varies between pale pink and the normal mucosal color. A strong family history has been suggested by a number of authors^24^, ^25^, ^50^.

Viral etiology has been initially shown by immunohistochemistry and later on by hybridization techniques identifying HPV 13 and 32, which were detected in 75-100% of the cases^32^.

HPV 13 and 32 were considered specific in focal epithelial hyperplasia, however, HPV 32 was also found in other oral lesions, but never outside the oral cavity^36^, ^39^, ^40^.

The studies and their results are depicted on table 3.

**Table 3:** Results from various authors as to HPV identification in the oral cavity and oropharynx focal epithelial hyperplasia.

Year	Investigator	Method	#.P/#.C	%	HPV type
1984	Syrjänen^27^	IHQ	1/1	(100)	HPV +
1987	Hernandez-Jauregui et al.^35^	SB	7/7	(100)	13
1989	Garlick et al.^37^	SB	4/4	(100)	13(75%), 32(25%)
1989	Henke et al.^38^	ISH	16/17	(95)	13(59%), 32(35%)
1991	Padayachee & Van Wyk^36^	IHQ	7/18	(39)	HPV +
		ISH	15/18	(83)	32 (60%), 13(33%)
					11 (7%)
2002	Schwenger et al.^76^	PCR	5/5	(100)	13(20%) e 32(60%)

#= number; P = positives; C = cases; IHQ = immunohistochemistry; ISH = in situ hybridization; SB = Southern blot hybridization; PCR = polymerase chain reaction; HPV + = positive human papillomavirus.

### Human papillomavirus prevalence in oral cancer and oropharynx

Unfortunately, oral cancer still bears high mortality rates. Its incidence rate varies from one region to another, being high in India, Sri Lanka, Vietnam, Filipinas, Hong Kong and Taiwan, where about 30% of all the cancers occur in the oropharynx. India has approximately 56,000 new cases per year, and is probably among the highest incidence rates in the world^41^.

It is a disease that affects patients in their 50th decade of life, being more evident between 60 and 70 years of age. Oral cancer includes malignant neoplasms in the lips, oral cavity and oropharynx^42^. Oral cancer is a severe and growing public health concern in Brazil, corresponding to 4% of all cancer types, occupying the eighth place among the tumors that affect men, and the 11th among women^43^.

The most common location for oropharynx cancers, is the palatine tonsils. Notwithstanding, lingual cancer corresponds to 30% of these^44^. About 90% of these lesions are located in soft tissues and are originated from squamous epithelium^20^.

Cigarette smoking and alcohol are the main causes of oral cancer. They act in synergy and are a dose-dependent^12^, ^17^. However, part of the population develops oral cancer without having been exposed to these risk factors, suggesting other causes, such as: genetic predisposition, diet and viral agents, most specifically HPV 345.

The simultaneous presence of chemical agents and HPV infection in the oral mucosa may favor the malignant transformation^46^. Notwithstanding, the HPV role as an etiological agent in the oral cancer is less than that from alcohol and tobacco use because HPV infection prevalence is less than alcohol and tobacco use in the genesis of oral cancer^20^, ^47^.

Syrjänen et al.^48^, in 1983, suggested that HPV may be involved in oral cavity squamous cell carcinoma, when they described HPV cytopathological alteration in oral cancer, identical to those previously found in uterine cervix cancer, although we still lack proof by other DNA hybridization methods.

Many studies have shown HPV 16 to be the most prevalent type in oral cancer, as it happens in anogenital cancer^24^, ^25^, ^49^.

In a 2001 metanalysis, Miller & Johnstone^47^, showed an HPV increase in oral epithelial carcinoma and dysplasias when compared to the normal oral mucosa, mainly in high risk genotypes. Results show HPV to be an independent risk factor for squamous cell oral carcinoma. In 2003, Herrero et al.^50^, studied HPV in oral cancer and observed a higher prevalence of HPV 16 in the oral cavity and the oropharynx among patients that had more than one sex partner and/or who practiced oral sex, while the lowest prevalence was seen among smokers.

According to many studies, the HPV prevalence rate in oral cancer varied from 0-100%^17^, ^25^, ^29^, ^51^, ^52^, ^53^, ^54^. This broad variation in HPV detection rate is explained by the different detection methods used in the HPV investigation^25^.

One of the greatest difficulties in identifying HPV in oral cancer patients is the presence of this virus in only one subpopulation of cells and the reduced number of copies of these cells which are infected. That is why we need high sensitivity detection methods^55^.

### Verrucous carcinoma

The verrucous carcinoma was first described as a variant of the squamous cells carcinoma, which originates in the oral cavity.

Also known as Ackerman’s tumor, its growth is exophytic, slow and invasive -only superficially, with a low metastasis rate, and it may be treated with simple excision. Many authors have reported the HPV presence in the verrucous carcinoma^20^, ^32^, ^46^, ^56^, ^57^, ^58^.

### Squamous cells carcinoma (SCC)

Encompasses about 95% of all the oral cancers. Its clinical aspects vary from a nodule up to a chronic ulcer. Many researchers have shown HPV present in SCC^18^, ^59^, ^60^, ^61^, ^62^. Initially we did microscopy, immunohistochemistry and, more recently, molecular biology tests^20^.

Studies have shown the important role of HPV in head and neck cancers, and also suggest that HPV 16 may be involved in the development of some oral carcinomas^56^, ^60^, ^63^.

Table 4 shows oral carcinoma studies with their results and techniques.

**Table 4:** Results from various authors as to HPV identification in oral carcinoma.

Year	Investigator	Lesion	Method	#. P/#. C	%	HPV type
1985	De Villiers et al.^75^	CCE	SB	3/7	(43)	2, 16
1986	Syrjnänen^68^	CCE	ISH	1/2	(50)	16
			IHQ	0/2	0	-
1991	Zeuss et al.^29^	CCE	ISH	0/15	0	-
1991	Young et al.^54^	CCE	ISH	0/17	0	-
		CV	ISH	0/10	0	-
1993	Holladay & Gerald.^73^	CCE	PCR	7/37	(19)	16e 18
1993	Noble-Topham et al.^57^	CV	PCR	12/25	(48)	6,11(10%), 16(20%)
						18(84%)
1994	Ostwald et al.^61^	CCE	PCR/SB	16/26	(62)	16(45%), 18(35%)
						6,11 (15%)
1995	Balaram et al.^46^	CCE	PCR	67/91	(74)	6(13%), 11(20%),
						16(42%), 18(47%)
		CV	PCR	10/15	(67)	6(10%), 11(20%), 16, 18(60%)
1996	González-Moles et al.^72^		CCE	PCR	11/37	(30)
1996	Snijders et al.^53^	CCE	PCR	7/32	(22)	16
1996	Cruz et al.^71^	CCE	PCR	19/35	(55)	16(79%), nd (21%)
1998	Mineta et al.^70^	CCE	PCR	3/14	(21)	16
1998	Wilczynski et al.^62^	CCE	PCR	14/21	(64)	16(80%), 33(10%),
						59(10%)
1998	Premoli-de-Percoco et al.^69^	CCE	ISH	35/50	(70)	16 e 18 (80%), 16, 18, 6 e 11 (20%)
1998	Miguel et al.^60^	CCE	PCR	2/27	(8)	16
1998	Elamin et al.^55^	CCE	PCR	14/28	(50)	16(43%),
						16 e 6(36%), 6(21%)
1998	D’Costa et al.^68^	CCE	PCR	15/100	(15)	
1998	Koh et al.^67^	CCE	PCR/SB	22/42	(52)	16(68%), 18 (27.5%)
						33(18.5%)
1999	Bustos et al.56	CCE (3/9)	ISH	09/33	(27)	16
		CV (5/9)				
		Melanoma (1/9)				
2002	Kojima et al.18	CCE	PCR	35/53	(66)	38
2003	Sugiyama et al.63	CCE	PCR	30/86	(35)	16

#= number; P = positives; C = cases; IHQ = immunohistochemistry; ISH =in situ hybridization; SB = Southern blot hybridization; PCR = polymerase chain reaction; nd= undetermined; CV= verrucous carcinoma; CCE= squamous cell carcinoma.

## MATERIALS AND METHODS

We searched the MEDLINE database and textbooks, English and Portuguese medical literature, from January 1990 to December 2003, and found reports of HPV prevalence by virus detection methods (immunohistochemistry and molecular biology exams) in the normal oral mucosa, in virus-related benign lesions (squamous cells papilloma, condiloma, verruca and focal epithelial hyperplasia) and in oral cancer.

In the MEDLINE database, we used the following keywords: detection, human papillomavirus oral, Polymerase Chain Reaction, human papillomavirus, oral cavity, normal oral cavity, oral lesions, papillary lesions, oral condiloma acuminata, oral warts, oral focal epithelial hyperplasia, oral cancer, squamous cell carcinomas, among others, they were used alone or in combination in the search.

After we selected these scientific papers we included some relevant papers about oral HPV, published before the period established in the search string.

## DISCUSSION

HPV prevalence in the oral cavity and oropharynx is considered uncertain, because many studies have shown different results with the estimate of a small number of patients, and a modest identification among the different types of HPV found in mucosal lesions^17^.

HPV diagnosis in the oral mucosa and the oropharynx may be suspected by the clinical exam of the lesion, cytology and biopsy; however the molecular biology exams are the ones able to detect the HPV DNA in the cell, stressing the polymerase chain reaction as the most sensitive method to detect HPV. Notwithstanding, the assessment of different methods used to detect HPV16 is important and fundamental in order to establish the etiological role of HPV in oral lesions.

Table 1 shows HPV 16 prevalence in the normal oral mucosa in 16 results from 14 authors, by the SB, ISH and PCR detection methods, making up 56% of all the results, and this seems to depend on the population examined and the choice of method used to detect HPV^21^, ^16^.

Still table 1 shows 6 negative results obtained by the ISH, SB and PCR detection methods, which make up 40% of the total number of results investigated.

It is probably due to the small number of samples used in the research and the very difficulty in detecting HPV in swabs and biopsy samples.

Among the factors that generate controversy about HPV prevalence in the normal oral mucosa seen on Table 1, we can stress the large variation in HPV detection rates, from 0% to 100% despite the use of more sensitive methods -such as PCR; another factor is the relationship between results and sample sizes, which varied in 3 samples, with 100% positiveness for HPV in the Tominaga et al.^64^ study from 1996, reaching up to 212 samples with 15% of HPV detection in the Kellokoski et al.^65^ study from 1992, thus showing a great difference between the results, which is probably due to a failure in the HPV detection methods or in harvesting the material or, even, because we do not know the exact HPV infection pathway in the normal oral mucosa^32^, ^13^, ^16^.

The human papillomavirus infection in oral cavity and oropharynx benign lesions (squamous cell papilloma, condilomas and verruca) in 25 results from 14 authors was of HPV 6 and 11, in papillomas and in condilomas, through the following methods: IHQ, ISH and PCR; in oral warts, the prevalence was of 2 and 57, by the IHQ, ISH, HSB and PCR methods, with HPV 2 dominance. The presence of HPV is clear in all cases, varying from 13% to 100% in positiveness, with its prevalent types, according to the lesion, regardless of the methods used. It is very likely that this happens because these lesions bear a larger number of cells with HPV DNA. We could also see a dominance of low risk HPV, specially HPV 6 and 11 justified because they are the types most frequently associated with oral benign papillomatosis lesions^29^, whilst the high risk ones, like the HPV 16, only showed up in 3 results.

Table 3 depicts HPV prevalence in oral focal epithelial hyperplasia in 7 results from 6 authors and it was HPV 13 and 32, through the many detection methods: IHQ, ISH, HSB and PCR, with a variation in HPV detection rates of 39% to 100%. We could also see that all results were HPV positive and in 2 cases the HPV was not typed, justified by the IHQ technique, what makes HPV evident in this disease.

Table 4 depicts a variation in HPV detection rates, from 0% to 74% in 24 results of oral cancer from 21 authors: it is probably due to the different detection methods bearing different sensitivity and sample sizes used in the research, from 2 to 100 samples. Another factor is the difficulty in detecting HPV in oral cancer, due to the presence of such virus in only one subpopulation of cells and the low number of copies found in infected cells^55^.

Still in Table 4, we see a high HPV 16 prevalence through the ISH, HSB and PCR techniques, being present in 80% of all the results. HPV 16 rates detected varied in 8%, in other words, 2 positive results from the 27 samples in the Wilczynski et al.^62^ (1998) study, and this suggests a huge discrepancy among the results. We could also see 4 negative results for HPV by the IHQ and ISH methods, which is probably due to the low sensitivity of these methods when compared to PCR and the small sample size. Therefore, we still require more studies using different HPV detection methods to better confirm the virus presence in oral cancer.

HPV 16 presence in oral cancer in many studies depicted in Table 4 does not prove that the virus be indeed responsible for the disease, notwithstanding, it does prove that it may contribute to the incidence of oral cancer, specially among non-smokers and non-alcoholics^3^, ^45^, or even in the simultaneous presence of chemical agents and HPV infection, which may lead to malignant transformation.

Other factors that contribute to increase HPV prevalence in the oral cavity and oropharynx are: a reduction in the patient’s immune response for the virus^66^, having more than one sexual partner and the practice of oral sex^50^ - these all increase the likelihood of an HPV infection and its recurrence.

HPV 16 prevalence found in the oral cancer results (Table 4) and the normal oral mucosa (Table 1) suggests that HPV 16 is the one most prevalent in the oral mucosa and oropharynx. Comparing HPV prevalence between tables 1 and 4 could value the HPV relationship in the genesis of certain dysplasia. Notwithstanding, the results seen in the tables bring about controversies, which are attributed, mainly to the sensitivity variation of the methods employed, as well as the diversity of the populations studied and the sample sizes.

## CONCLUSION

The literature analysis on the HPV prevalence in the oral cavity and oropharynx leads us to the following conclusions:

1. Among the techniques used to diagnose HPV, the most sensitive is PCR;
2. HPV prevalence in the normal oral mucosa presents discrepant results;
3. HPV prevalence in virus-related benign lesions is confirmed;
4. Despite HPV prevalence in oral cancer there still are controversies as to the virus presence and oral carcinogenesis;
5. Thus, more studies are necessary in order to enhance the methods used to detect HPV DNA and the sample collection techniques (swabs or biopsies), aiming at having less result interference and greater clarifications about HPV infection and its prevalence in both the oral cavity and the oropharynx, and we are motivated to continue the HPV research, especially in the normal oral mucosa and in oral cancer.
